# Copy Number Variation Analysis on a Non-Hodgkin Lymphoma Case-Control Study Identifies an 11q25 Duplication Associated with Diffuse Large B-Cell Lymphoma

**DOI:** 10.1371/journal.pone.0105382

**Published:** 2014-08-18

**Authors:** Lucia Conde, Jacques Riby, Jianqing Zhang, Paige M. Bracci, Christine F. Skibola

**Affiliations:** 1 Department of Epidemiology, School of Public Health and Comprehensive Cancer Center, University of Alabama at Birmingham, Birmingham, Alabama, United States of America; 2 Department of Epidemiology and Biostatistics, University of California San Francisco, San Francisco, California, United States of America; The Ohio State University, United States of America

## Abstract

Recent GWAS have identified several susceptibility loci for NHL. Despite these successes, much of the heritable variation in NHL risk remains to be explained. Common copy-number variants are important genomic sources of variability, and hence a potential source to explain part of this missing heritability. In this study, we carried out a CNV analysis using GWAS data from 681 NHL cases and 749 controls to explore the relationship between common structural variation and lymphoma susceptibility. Here we found a novel association with diffuse large B-cell lymphoma (DLBCL) risk involving a partial duplication of the C-terminus region of the *LOC283177* long non-coding RNA that was further confirmed by quantitative PCR. For chronic lymphocytic leukemia/small lymphocytic lymphoma (CLL/SLL), known somatic deletions were identified on chromosomes 13q14, 11q22-23, 14q32 and 22q11.22. Our study shows that GWAS data can be used to identify germline CNVs associated with disease risk for DLBCL and somatic CNVs for CLL/SLL.

## Introduction

Non-Hodgkin lymphoma (NHL) is a common malignancy of the lymphoid system that encompasses a heterogenous spectrum of diseases, with different clinical, pathological and morphological characteristics. The most common NHL subtypes are diffuse large B-cell lymphoma (DLBCL), follicular lymphoma (FL), and chronic lymphocytic leukemia/small lymphocytic lymphoma (CLL/SLL), which account for approximately 33%, 20%, and 5–10%, respectively, of all lymphomas in the United States [1]. Despite the successes of recent genome-wide association studies (GWAS) in the identification of novel NHL loci [2]–[7], much of the heritable variation in NHL risk remains to be explained, and it is likely that structural variants other than SNPs might account for some of this missing heritability.

Copy number variants (CNVs), detected through molecular cytogenetic techniques or high-density SNP arrays, have been associated with numerous diseases including several lymphoma subtypes. Known recurrent aberrations have been found in ∼80% of CLL patients [8], with deletions in chromosomes 13q14, 17p13, 11q22-23, 6q and trisomy 12 being the most frequent [8]. CNV studies using DLBCL tumor tissue revealed that DLBCL subgroups could be segregated by the frequency of particular somatic chromosomal aberrations [9]–[10]. Thus, aberrations most characteristic of the activated B-cell like (ABC) DLBCL subtype, which has a poor clinical outcome, include trisomy 3, gains of 3q and 18q21–q22, and deletions of 6q21–q22 and the INK4a/ARF locus on chromosome 9, whereas the germinal center B-cell (GCB) subtype, which is more common in younger adult DLBCL cases and has a better clinical outcome, exhibit frequent amplifications of 12q12, the *MIHG1* locus on chromosome 13, the *REL* locus on chromosome 2 and deletion of *PTEN* on chromosome 10 [9]–[10]. For FL, recurrent copy number alterations have been observed in chromosomes 1, 5–8, 10, 12, 17–19 and 22, some of them correlating with lower survival and/or risk of transformation from FL to DLBCL [11]. Whereas the presence of somatically acquired structural variants in DLBCL and FL has been previously investigated, the role of germline structural variants in NHL susceptibility is relatively unexplored and their contribution to lymphoma risk remains unclear. Although existing tools based on SNP array data have lower sensitivity to detect CNVs than standard laboratory approaches such as multiplex ligation-dependent probe amplification [12], the use of SNP arrays for CNV discovery and detection has several advantages such as being a cost-effective and requiring less sample per experiment compared to other techniques such as CGH arrays [13]. Thus, numerous studies including those in colorectal cancer [14], testicular germ cell cancer [15], and breast and ovarian cancer [16] have successfully used GWAS data to identify CNVs that are associated with disease risk.

Here, we extended our previous GWAS SNP analysis to report the results of a genome-wide CNV analysis in 681 NHL cases and 749 controls from the San Francisco Bay Area. In this study, we investigated the role of germline structural variants in risk of DLBCL and FL, and we also sought to determine if we could detect the presence of somatic structural variants in CLL/SLL using GWAS data generated from blood DNA.

## Methods

### Study Population

A population-based case-control study of NHL (2,055 cases, 2,081 controls) that included incident cases diagnosed from 2001 through 2006 was conducted in the San Francisco Bay Area. Details of the study design and methods have been described previously [3]. Briefly, eligible patients were identified through the cancer registry and met the following criteria at diagnosis: aged 20–85 years, resident of one of the six Bay Area counties and able to complete an in-person interview in English. Controls were identified by random digit dial and random sampling of Center for Medicare and Medicaid lists, met the same eligibility criteria as cases with the exception of NHL diagnosis, and were frequency-matched to patients by age in five-year age groups, sex and county of residence. Blood and/or buccal specimens were collected from eligible cases and controls that participated in the laboratory portion of the study (participation rates, 87% and 89%, respectively). To confirm NHL diagnosis and for consistent classification of NHL subtypes using the WHO classification, the study’s expert hematopathologist re-reviewed patient diagnostic pathology materials (including diagnostic slides, pathology, immunohistochemistry and flow cytometry reports) for >98% of consenting cases, with review of diagnostic slides in addition to pathology reports conducted for 54% of cases. Approximately 23% of NHL subtypes were reclassified, and approximately 1% of cases were dropped as not NHL after expert re-review. The U.C. San Francisco Committee on Human Research and the U.C. Berkeley Committee for Protection of Human Subjects approved study protocols. All study participants provided written informed consent prior to interview and biospecimen collection.

### Genotyping

Details of the genotyping and quality control have been published previously [3]. Briefly, DNA from 1,577 study participants was genotyped using Illumina HumanCNV370-Duo BeadChip (Illumina, San Diego, CA), which comprises over 370,000 markers, including over 14,000 CNV regions. Genotype clustering was conducted with Illumina Beadstudio software from data files created by an Illumina BeadArray reader. Individuals with call rates <95% were excluded from analysis. We checked population stratification using multidimensional scaling (MDS) as described previously [3]. Specifically, we first merged our data with genotypes from 209 unrelated HapMap Phase II individuals from the CEU, YRI and JPT+CHB panels and we selected a subgroup of 33,838 unlinked SNPs, by pruning those with r2>0.1 using 50-SNP windows shifted at 5-SNP intervals. We ran the MDS analysis on the matrix of IBS pairwise distances and selected 100 as the number of dimensions to be extracted. Samples with evidence of non-European ancestry were identified by inspection of the MDS plots and excluded from analysis regardless of their self-reported origin, resulting in a final dataset of 681 NHL cases (213 FL, 257 DLBCL, 211 CLL/SLL [180 untreated and 31 treated]) and 749 controls that were used for CNV analysis. Among the 211 CLL/SLL samples, 180 were obtained from patients that did not receive any treatment and that are referred hereafter as CLL/SLL samples, whereas the remaining 31 were samples obtained during or after chemotherapy, and therefore were analyzed separately and referred hereafter as treated CLL/SLL samples.

### CNV Calling and Analysis

We used the software PennCNV [17] to call and analyze CNVs. PennCNV implements a hidden Markov model (HMM) that combines total and allelic signal intensity data obtained for each marker on the genotyping array, with SNP population allele frequency to generate CNV calls. Signal intensity data in the form of log R ratio (LRR) and B allele frequency (BAF) values were obtained directly from the Beadstudio software, and the HMM and the population frequency of the B allele were obtained from PennCNV. Quality control filtering was used at a sample level to exclude unreliable samples. This included removing samples with LRR standard deviation (LRR_SD) >0.3, B allele frequency drift (BAF_DRIFT) >0.01, or waviness factor (WF) outside of a −0.05,0.05 range. Individuals with more than 100 CNVs were considered low quality samples and were also eliminated from analysis. A total of 62 NHL cases and 19 controls were excluded in this filtering step. Centromeres and telomeres are known to harbor spurious CNV calls, so coordinates for these regions were downloaded from the UCSC genome browser build 36 (hg18) (http://genome.ucsc.edu/) and individual CNV calls that overlapped 50% or more with these regions were excluded from analysis. Although immunoglobulin regions are also prone to contain spurious CNV calls, we decided to not exclude them due to the significance of immunoglobulin genes in NHL. Finally, CNVs with confidence score <10, length <1 kb or expanding less than 5 markers were also discarded. CNV calls were considered novel if they did not overlap with any of the copy number and Indel loci catalogued in the Database of Genomic Variants, build 36 (hg18) (DGV, http://projects.tcag.ca/variation/). The UCSC Genome Browser was used to map CNVs to genes, and CNVs were assigned to cytobands if they overlapped 50% or more with the cytoband boundaries. A Fisher's exact test was used to evaluate the association of CNVs with NHL, and Fisher p-values were adjusted for multiple comparisons by FDR using the p. adjust function in R. Signal intensity data (LRR and BAF values) have been deposited in the NCBI’s Gene Expression Omnibus database (http://www.ncbi.nlm.nih.gov/geo/) and are accessible through GEO Series accession number GSE58718.

### CNV Molecular Confirmation

Copy number verification of the duplication observed in 11q25 for DLBCL was performed using quantitative PCR on the 11 DLBCL cases that presented the duplication and 11 random controls with no duplication in the region. Nine primer pairs were designed to amplify regions covering the full length of LOC283177, from the C-terminus on the right to the N-terminus on the left and referred to as P1 to P9 (**Table S1**), using NCBI’s primer designing tool Primer-Blast (http://www.ncbi.nlm.nih.gov/tools/primer-blast/). The housekeeping gene GAPDH (forward 5′-GTGAAGGTCGGAGTCAACG-3′, reverse 5′-TGAGGTCAATGAAGGGGTC-3′) was used as the reference gene for normalization of possible variations of DNA concentration and difference in DNA quality between subjects. Real-time quantitative PCR (qPCR) was done with SYBR-Green BioRad Supermix II on a CFX-Connect instrument (BioRad, Hercules, CA). Raw qPCR data was obtained as Cq values for each primer pair minus Cq value for GAPDH for each subject (ΔCq). For each individual primer pair, Δ.ΔC represents the difference between the average ΔCq of the 11 cases and the average ΔCq of the 11 controls. Relative abundance, or CNV, is calculated using the formula 1/2^Δ.ΔCq^ in which a difference of 1 Δ.ΔCq is the result of a 2-fold difference in template copy numbers.

## Results

### CNV Discovery

After strict quality control filtering, 26,807 CNV calls were observed in 619 NHL cases (205 FL, 242 DLBCL, 148 CLL/SLL, 24 treated CLL/SLL) and 730 controls (**Table 1**).

**Table 1:** Summary of the copy number variation (CNV) analysis by lymphoma subtype.

	FL(n = 205)	DLBCL(n = 242)	CLL/SLL		Controls (n = 730)
			Untreated(n = 148)	Treated(n = 24)	
Samples with aberrations	205	242	148	24	730
Total CNVs	4243	4749	3304	466	14045
Homozygous deletions (CN = 0)	442 (10.4%)	502 (10.6%)	296 (9%)	41 (8.8%)	1477 (10.5%)
Hemizygous deletions (CN = 1)	2346 (55.3%)	2446 (51.5%)	1768 (53.5%)	250 (55.6%)	7449 (53.0%)
Duplications (CN = 3)	1443 (34%)	1791 (37.7%)	1228 (37.2%)	173 (37.1%)	5082 (36.2%)
Biallelic duplications (CN = 4)	12 (0.3%)	10 (0.2%)	12 (0.4%)	2 (0.4%)	37 (0.3%)
CNV calls per individual	20.7	19.6	22.3	19.4	19.2
Ratio of deletions/duplications	1.92	1.64	1.66	1.66	1.74
CNV length					
Average CNV size	82.5 kb	92.1 kb	303.1 kb	86.8 kb	82.0 kb
Range CNV size	1.0 kb–1.5 Mb	1.1 kb–20.2 Mb	1.0 kb–77.3 Mb	1.1 kb–1.0 Mb	1.0 kb −10.0 Mb
Average number of SNP per CNV call	12.8	13.4	42	13.7	12.6
Range number of SNPs per CNVcall	5–205	5–1382	5–9374	5–145	5–362

The average number of CNVs per individual in cases ranged from 19.5 CNVs/individual in treated CLL/SLL to 22.3 CNVs/individual in CLL/SLL, and it was slightly higher than the average number of CNVs per individual in the controls (19.2, **Table 1**). The average CNV size was markedly higher in CLL/SLL in comparison with the treated CLL/SLL cases, the FL and DLBCL subtypes, as well as the controls (303 kb in CLL/SLL versus 80–98 kb in treated CLL/SLL, FL and DLBCL, and 82 kb in controls, **Table 1**). These data suggested that the treated CLL/SLL samples have a CNV content more similar to FL and DLBCL than to the CLL/SLL samples. Based on the database of Genomic Variants, 9.4%, 8.2%, 7.5%, and 7.4% of the detected CNVs in CLL/SLL, treated CLL/SLL, FL and DLBCL, respectively are novel, whereas only 7.0% of the CNVs in the controls were novel.

### DLBCL

**Figure 1:**
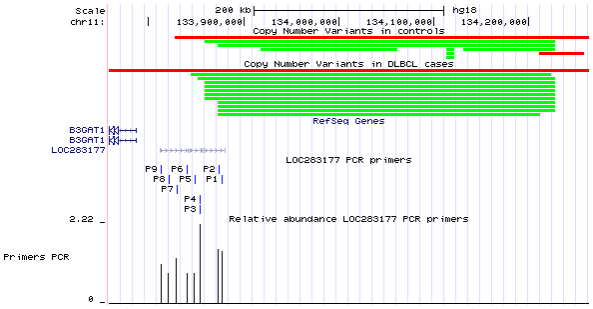
The figure shows, on the top, the aberrations (deletions in red, duplications in green) found in the 11q25 region in controls and DLBCL cases after CNV analysis using a genotyping array. On the bottom, the figure shows the locations of the 9 PCR primers designed to cover the LOC283177 gene. qPCR confirmed the partial duplication of LOC283177 (P = 0.004), and the region of breakpoint was determined to be located between primers P4 and P5. Coordinates are shown with respect to the NCBI36/hg18 assembly.

A significant association was found for duplications in 11q25 and DLBCL (**Table S2** and **Figure S1**), where 15 (6.2%) of the DLBCL cases showed duplications versus 6 (1.1%) of the controls. Twelve out of the 15 DLBCL cases with duplications in 11q25 shared a common duplication of approximately 340 Kb located 86 kb upstream of *B3GAT1*, a member of the glucoronyltransferase gene family whose gene product functions in the biosynthesis of the carbohydrate epitope *HNK-1* (human natural killer-1, also known as *DC57* and *LEU7*). The only gene in the region of the duplication is *LOC283177*, a long non-coding RNA (lncRNA) gene that was partially duplicated in 11 (4.5%) of the DLBCL samples (**Figure 1**). *LOC283177* was therefore the most significantly associated gene with DLBCL (P_FDR_ = 2.77×10^−2^), followed by *DGCR6* and *PRODH*, both located on chromosome 22q11.21 and duplicated in 10 (4.1%) of the DLBCL cases, although the association for these genes was not significant after correction for multiple testing. Although no cytobands were significantly associated with deletions in DLBCL, we observed that a deletion of the pseudogene *ZNF826P* in 19p12 was approaching significance (P_FDR_ = 7.84×10^−2^).

When we stratified the DLBCL cases by age, we found that, although non-significant after correction, the 11q25 duplication was more strongly associated with the young group (<60yo, n = 95, P = 7.64×10^−4^), which generally corresponds to the GCB subtype, than with the older DLBCL group (> = 60, n = 144, P = 1.79×10^−3^). Moreover, although the number of cases was lower, the association of *LOC283177* duplications with DLBCL was stronger in the younger group (P_FDR_  = 2.07×10^−2^) than in the combined DLBCL cohort.

To verify the partial duplication of *LOC283177*, we performed qPCR on the 11 DLBCL samples that presented the duplication and 11 random controls with no duplication in the region. The PCR data for the nine primers pairs designed to cover the gene showed that the relative abundance of the four primers on the C-terminus were, on average, 1.8-fold in the DLBCL cases compared to the controls whereas the other 5 primers on the N-terminus showed no evidence of duplication (average 0.98-fold, P = 0.004), confirming the results from the CNV analysis that suggested a partial duplication of *LOC283177* in DLBCL (**Figure 1**).

### FL

We did not find any CNV significantly associated with FL at an adjusted FDR p-value<0.05. The strongest association (P = 7.27×10^−4^) was found for deletions of chr3q13.31, where 12 (5.9%) FL cases presented deletions versus 10 (1.4%) of the controls (**Table S3**). Among all the genes, *ZNF658* in chromosome 9 was the one most strongly associated with aberrations in FL, with 7 (3.4%) of the FL cases presenting duplications in this gene (P = 5.35×10^−4^).

### CLL/SLL and treated CLL/SLL

Among the known CLL/SLL recurrent deletions, statistically significant associations were observed for deletions of chromosomes 13q14, 11q22-23 and 14q32 in our study (**Table S4**), whereas no significant associations were found for 6q and 17p deletions and our CLL/SLL cases. In agreement with previous studies, del(13q14) was the most significant aberration observed in our CLL/SLL cases (**Table S4** and **Figure S2**). Among the 31 (20.9%) CLL/SLL cases that presented deletions in this region, 25 of them had del(13q14) as the sole abnormality, 4 cases presented also del(11q) and 2 del(17p). The next strongest associated aberrations were deletions of 14q32 (**Table S4**), which were observed in 36 (24.3%) of the CLL/SLL cases, followed by deletions of 11q22-23 (**Table S4**). Additional statistically significant associations were found in chromosome 22q11.22 (**Table S4**), where deletions associated with CLL in the region were previously found to be related to the rearrangement of the immunoglubulin lambda chain locus [18]. As expected, the genes most frequently deleted were located in these cytobands (**Table S5**). Thus, members of the DLEU family, *ST13P4* and the miRNAs, MIR16-1 and MIR15, all located in 13q14 were the most commonly deleted genes in CLL/SLL, with *DLUE2* being deleted in 27 (18.2%) of the CLL/SLL cases (P_FDR_ = 2.24×10^−17^). Significant gene deletions were observed also for the pseudogene *ADAM6* in 14q32 (P_FDR_ = 2.08×10^−5^), the miRNA MIR650 in 22q11.22 (P_FDR_ = 5.27×10^−4^), and a cluster of genes in 11q22-23 (6.27×10^−3^>P_FDR_<2.46×10^−2^). Although some duplications were observed at a nominal p-value<0.05, none of these remained significant after correction.

Similarly, in the treated CLL/SLL group (n = 24), some associations were observed that were significant before correction, with deletions in 14q32 being the most significantly associated aberration (P = 5.87×10^−3^), although it did not remain significant after adjustment for multiple comparisons. Of note, as opposed to the CLL/SLL group, no deletions were observed in 13q14, 11q22-23 or 22q11.22 in any of the treated CLL/SLL cases.

## Discussion

In this study we carried out a CNV analysis of 681 NHL cases and 749 controls to explore the relationship between common structural variation and lymphoma risk. A significant association in 11q25 was observed for DLBCL, where we found a partial duplication of the C-terminus region of the *LOC283177* lncRNA that was further confirmed by qPCR. Although no previous association of this uncharacterized gene with DLBCL has been previously reported, a duplication in the region overlapping *LOC283177* has been recently described in a CNV study of acute myeloid leukemia [19]. Most lncRNAs remain uncharacterized, but a significant number have been shown to exhibit cell-specific expression and association with human diseases, with several lncRNAs being dysregulated in various diseases, especially cancer [20]. Whereas the biological relevance of *LOC283177* in DLBCL needs further investigation, our results suggest that this lncRNA could be a potential susceptibility locus for DLBCL. Interestingly, the association of *LOC283177* with DLBCL was slightly higher in the younger DLBCL group than in the whole DLBCL cohort. Although this suggests that the duplication might be more characteristic of the GCB subtype, further studies with confirmed pathology would be needed to support this observation. In the FL subtype, deletions of chr3q13.31, a region associated with CNVs in osteosarcoma [21] and frequently deleted in human cancers [22], were observed in 5.9% of the FL cases, although the association did not remain significant after multiple testing correction. Further investigation of the association of chr3q13.31 deletions with FL risk may be warranted. On the other hand, in agreement with previous studies, strong associations were observed for CLL/SLL and deletions on chromosomes 13q14, 11q22-23, 14q32 and 22q11.22. Although our analysis focused on germline structural variants, it is not uncommon to observe the presence of these somatic acquired structural variants in blood from CLL/SLL patients, due to the high content of circulating tumor cells present in CLL/SLL. Of note, with exception of 14q32, none of the treated CLL/SLL samples presented deletions in these regions, although this could be a power issue due to the low number of samples analyzed or due to the somatic nature of these aberrations, which are expected to be less common in CLL/SLL patients undergoing treatment.

Although the sample size of our study is small and the genotyping platform used was a low density SNP array, we were able to identify with high statistical significance the most common established somatic aberrations in the CLL/SLL subtype, as well as a novel duplication in germline DNA of DLBCL cases, further confirmed by qPCR, suggesting the validity of our approach and the potential role of germline CNVs as NHL susceptibility loci. Nonetheless, our findings will require further validation in independent studies. Additionally, it is possible that our study might be underpowered to detect CNVs of lower frequencies, and larger sample sizes are necessary to further investigate the effects of CNVs in lymphoma susceptibility.

## Supporting Information

Figure S1 **CNV results for DLBCL cases and controls in the 11q25 chromosomal region.** Deletions and duplications in the region are shown in red and green respectively. Coordinates are shown with respect to the NCBI36/hg18 assembly.
(DOC)

Figure S2 **CNV results for CLL/SLL cases and controls in the 13q14 chromosomal region.** Deletions and duplications in the region are shown in red and green respectively. Coordinates are shown with respect to the NCBI36/hg18 assembly.
(DOC)

Table S1 **Primers designed to amplify the LOC2831772 region on 11q25.**
(DOC)

Table S2 **Aberrations found in the DLBCL cases at nominal P<0.05.** Aberrations that remained significant after correction (P_FDR<0.05) are shown in bold.
(DOC)

Table S3 **Aberrations found in the FL cases at nominal P<0.05.** None of these aberrations remained significant after correction (P_FDR<0.05).
(DOC)

Table S4 **Aberrations found in the CLL/SLL cases at nominal P<0.05.** Aberrations that remained significant after correction (P_FDR<0.05) are shown in bold.
(DOC)

Table S5 **Genes significantly deleted in the CLL/SLL cases (FDR p-value<0.05).**
(DOC)
