# Supplementary figures and images for: Copy Number Variation Analysis on a Non-Hodgkin Lymphoma Case-Control Study Identifies an 11q25 Duplication Associated with Diffuse Large B-Cell Lymphoma
Source: PLoS One. 2014 Aug 18;9(8):e105382. doi: 10.1371/journal.pone.0105382 (PMC4136881; doi:10.1371/journal.pone.0105382)

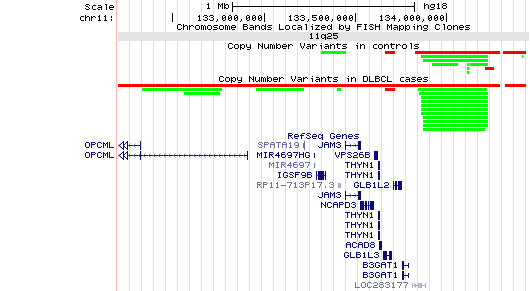

Supplement: Figure S1 — CNV results for DLBCL cases and controls in the 11q25 chromosomal region. Deletions and duplications in the region are shown in red and green respectively. Coordinates are shown with respect to the NCBI36/hg18 assembly. (DOC) [file pone.0105382.s001.doc]

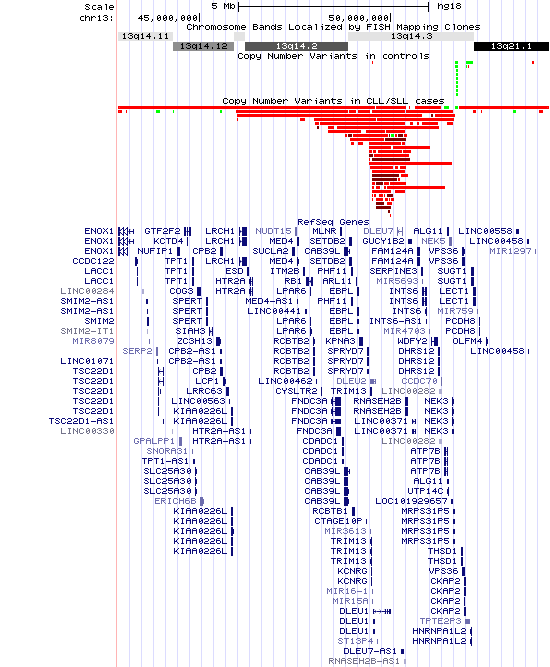

Supplement: Figure S2 — CNV results for CLL/SLL cases and controls in the 13q14 chromosomal region. Deletions and duplications in the region are shown in red and green respectively. Coordinates are shown with respect to the NCBI36/hg18 assembly. (DOC) [file pone.0105382.s002.doc]
