# Supplementary material for: Copy Number Variation Analysis on a Non-Hodgkin Lymphoma Case-Control Study Identifies an 11q25 Duplication Associated with Diffuse Large B-Cell Lymphoma
Source: PLoS One. 2014 Aug 18;9(8):e105382. doi: 10.1371/journal.pone.0105382 (PMC4136881; doi:10.1371/journal.pone.0105382)
Supplement: Table S1 — Primers designed to amplify the LOC2831772 region on 11q25. (DOC) [file pone.0105382.s003.doc]

| Primer location with respect to LOC283177 | Chromosomal location (NCBI37/hg19) | Primer Pair name | Primer Sequence | Amplicon length (bp) |
| --- | --- | --- | --- | --- |
| Primer Pair 1 (65522-65541: 65612-65593) |  | P1 |  | 91 |
| P1f | 134371888 - 13471907 |  | CCTGCTGTGGTAGGGAACTG |  |
| P1r | 13471978 - 13471959 |  | AAGTCCCTCCTAGGCTCTGG |  |
| Primer Pair 2 (62122-62141: 62314-62295) |  | P2 |  | 193 |
| P2f | 134368488 - 134368507 |  | GCTCTGAGCCCAGACAGTTT |  |
| P2r | 134368680 - 134368661 |  | GAGCCCCACTGGGAACTAAC |  |
| Primer Pair 3 (42719-42738: 42903-42884) |  | P3 |  | 185 |
| P3f | 134349085 - 134349104 |  | GGAGGTGTGAACCACATCGT |  |
| P3r | 134349269 - 134349250 |  | GAAAAGGCAACAGGCATGGG |  |
| Primer Pair 4 (42331-42350: 42464-42445) |  | P4 |  | 134 |
| P4f | 134348697 - 134348716 |  | TAGGGACAATGGAAACCGGC |  |
| P4r | 134348830 - 134348811 |  | GGCTGCAAAGATCCCTCCTT |  |
| Primer Pair 5 (36869-36888: 37053-37034) |  | P5 |  | 185 |
| P5f | 134343235 - 134343254 |  | TTTGGCCCTGCGTGGATAAT |  |
| P5r | 134343419 - 134343400 |  | GCATGGAGCCAGCTACTAGG |  |
| Primer Pair 6 (28873-28892: 29060-29041) |  | P6 |  | 188 |
| P6f | 134335239 - 134335258 |  | GGAGTCCAGGATCCCTCCTT |  |
| P6r | 134335426 - 134335407 |  | GGCACTTACGGAATCCACCA |  |
| Primer Pair 7 (17931-17950: 18079-18060) |  | P7 |  | 149 |
| P7f | 134324297 - 134324316 |  | ACAGCAAGACCAGGCTACAC |  |
| P7r | 134324445 - 134324426 |  | AGCTAGTAGGAAGCCAGCCT |  |
| Primer Pair 8 (9684-9703: 9835-9816) |  | P8 |  | 152 |
| P8f | 134316050 - 134316069 |  | GACAGGTGTCGGCAGGTAAA |  |
| P8r | 134316201 - 134316182 |  | TGTTTGGCAGCATCGTCTCT |  |
| Primer Pair 9 (1593-1612: 1754-1735) |  | P9 |  | 162 |
| P9f | 134307959 - 134307978 |  | AACAGTTTTGATTCGCGGGC |  |
| P9r | 134308120 - 134308101 |  | AGTCCAGCTCCACAAAGCTC |  |
