# Supplementary material for: Copy Number Variation Analysis on a Non-Hodgkin Lymphoma Case-Control Study Identifies an 11q25 Duplication Associated with Diffuse Large B-Cell Lymphoma
Source: PLoS One. 2014 Aug 18;9(8):e105382. doi: 10.1371/journal.pone.0105382 (PMC4136881; doi:10.1371/journal.pone.0105382)
Supplement: Table S2 — Aberrations found in the DLBCL cases at nominal P<0.05. Aberrations that remained significant after correction (P_FDR<0.05) are shown in bold. (DOC) [file pone.0105382.s004.doc]

| **Deletions** | | | | | |
| --- | --- | --- | --- | --- | --- |
| Band | Loc (Mb, NCBI37/hg19) | Number of DLBCL cases (%) | Number of controls (%) | Fisher's p-value | FDR-adjusted p-value |
| chr1p21.3 | 94.5-99.4 | 3 (1.2%) | 1 (0.1%) | 4.98E-02 | 1 |
| chr5q21.1 | 97.3-102.8 | 1 (0.4%) | 20 (2.7%) | 3.75E-02 | 1 |
| chr6q11.1 | 60.5-63.4 | 2 (0.8%) | 25 (3.4%) | 3.95E-02 | 1 |
| chr12p13.2 | 10-12.6 | 3 (1.2%) | 1 (0.1%) | 4.98E-02 | 1 |
| chr17q21.32 | 41.9-44.8 | 7 (2.9%) | 3 (0.4%) | 3.26E-03 | 1 |
| **Duplications** | | | | | |
| Band | Loc (Mb, NCBI37/hg19) | Number of DLBCL cases (%) | Number of controls (%) | Fisher's p-value | FDR-adjusted p-value |
| chr2p25.3 | 0-4.3 | 3 (1.2%) | 1 (0.1%) | 4.98E-02 | 1.00E+00 |
| chr2p11.1 | 91-93.3 | 5 (2.1%) | 4 (0.5%) | 4.73E-02 | 1.00E+00 |
| chr2q24.1 | 154.6-15. 6 | 3 (1.2%) | 1 (0.1%) | 4.98E-02 | 1.00E+00 |
| chr10p11.21 | 34.5-38.8 | 5 (2.1%) | 2 (0.3%) | 1.24E-02 | 1.00E+00 |
| chr11q14.3 | 87.9-92.3 | 3 (1.2%) | 0 (0.0%) | 1.53E-02 | 1.00E+00 |
| **chr11q25** | **130.3-134.5** | **15 (6.2%)** | **8 (1.1%)** | **4.12E-05** | **3.55E-02** |
| chr19q12 | 30.2-37.1 | 8 (3.3%) | 9 (1.2%) | 4.53E-02 | 1.00E+00 |
