# Supplementary material for: Copy Number Variation Analysis on a Non-Hodgkin Lymphoma Case-Control Study Identifies an 11q25 Duplication Associated with Diffuse Large B-Cell Lymphoma
Source: PLoS One. 2014 Aug 18;9(8):e105382. doi: 10.1371/journal.pone.0105382 (PMC4136881; doi:10.1371/journal.pone.0105382)
Supplement: Table S3 — Aberrations found in the FL cases at nominal P<0.05. None of these aberrations remained significant after correction (P_FDR<0.05). (DOC) [file pone.0105382.s005.doc]

| **Deletions** | | | | | |
| --- | --- | --- | --- | --- | --- |
| Band | Loc (Mb, NCBI37/hg19) | Number of FL cases (%) | Number of controls (%) | Fisher's p-value | FDR-adjusted p-value |
| chr2p12 | 75.4-83.7 | 10 (4.9%) | 12 (1.6%) | 1.51E-02 | 1 |
| chr2q12.3 | 106.7-108.6 | 2 (1.0%) | 0 (0.0%) | 4.79E-02 | 1 |
| chr3q13.31 | 115-118.8 | 12 (5.9%) | 10 (1.4%) | 7.27E-04 | 0.445366667 |
| chr3q24 | 144.4-150.4 | 5 (2.4%) | 1 (0.1%) | 2.40E-03 | 0.445366667 |
| chr3q26.31 | 172.5-177.3 | 6 (2.9%) | 5 (0.7%) | 1.77E-02 | 1 |
| chr4p15.2 | 23.1-27.9 | 3 (1.5%) | 0 (0.0%) | 1.04E-02 | 0.814981818 |
| chr4q28.1 | 124-129.1 | 3 (1.5%) | 1 (0.1%) | 3.49E-02 | 1 |
| chr5p14.2 | 23.3-24.7 | 4 (2.0%) | 1 (0.1%) | 9.34E-03 | 0.814981818 |
| chr5q13.2 | 68.4-73.3 | 28 (13.7%) | 62 (8.5%) | 3.19E-02 | 1 |
| chr6p24.3 | 7-10.6 | 2 (1.0%) | 0 (0.0%) | 4.79E-02 | 1 |
| chr6p21.31 | 33.6-36.8 | 2 (1.0%) | 0 (0.0%) | 4.79E-02 | 1 |
| chr6q14.1 | 75.9-83.9 | 73 (35.6%) | 204 (27.9%) | 3.78E-02 | 1 |
| chr6q24.1 | 139.1-142.9 | 15 (7.3%) | 24 (3.3%) | 1.65E-02 | 1 |
| chr7p22.3 | 0-2.1 | 3 (1.5%) | 1 (0.1%) | 3.49E-02 | 1 |
| chr8p11.23 | 38.5-39.5 | 101 (49.3%) | 302 (41.4%) | 4.62E-02 | 1 |
| chr9q34.3 | 136.6-140.3 | 2 (1.0%) | 0 (0.0%) | 4.79E-02 | 1 |
| chr11p13 | 31-36.4 | 6 (2.9%) | 2 (0.3%) | 1.96E-03 | 0.445366667 |
| chr11q14.2 | 85.3-87.9 | 29 (14.1%) | 62 (8.5%) | 2.26E-02 | 1 |
| chr13q12.12 | 22.2-24.4 | 5 (2.4%) | 4 (0.5%) | 2.81E-02 | 1 |
| chr13q12.3 | 27.8-31.1 | 2 (1.0%) | 0 (0.0%) | 4.79E-02 | 1 |
| chr13q21.31 | 60.5-64.1 | 6 (2.9%) | 4 (0.5%) | 9.83E-03 | 0.814981818 |
| chr14q11.2 | 19.1-23.6 | 17 (8.3%) | 23 (3.2%) | 2.81E-03 | 0.445366667 |
| chr15q12 | 23.3-25.7 | 2 (1.0%) | 0 (0.0%) | 4.79E-02 | 1 |
| chr15q13.3 | 29-31.4 | 8 (3.9%) | 10 (1.4%) | 3.76E-02 | 1 |
| chr16p12.3 | 16.7-20.5 | 42 (20.5%) | 88 (12.1%) | 2.90E-03 | 0.445366667 |
| chr17q21.31 | 37.8-41.9 | 25 (12.2%) | 141 (19.3%) | 1.75E-02 | 1 |
| chr17q22 | 47.6-54.9 | 3 (1.5%) | 1 (0.1%) | 3.49E-02 | 1 |
| chr17q23.2 | 55.6-58.4 | 19 (9.3%) | 30 (4.1%) | 6.82E-03 | 0.814981818 |
| chr18q12.3 | 35.5-41.8 | 78 (38.0%) | 197 (27.0%) | 3.10E-03 | 0.445366667 |
| chr19q13.31 | 47.8-50 | 17 (8.3%) | 32 (4.4%) | 3.30E-02 | 1 |
| chr20q12 | 37.1-41.1 | 3 (1.5%) | 0 (0.0%) | 1.04E-02 | 0.814981818 |
| **Duplications** | | | | | |
| Band | Loc (Mb, NCBI37/hg19) | Number of FL cases (%) | Number of controls (%) | Fisher's p-value | FDR-adjusted p-value |
| chr1p36.21 | 12.6-16.1 | 8 (3.9%) | 6 (0.8%) | 4.13E-03 | 0.954665 |
| chr5p13.3 | 29.3-34.4 | 14 (6.8%) | 18 (2.5%) | 4.43E-03 | 0.954665 |
| chr6q25.3 | 155.6-160.9 | 2 (1.0%) | 0 (0.0%) | 4.79E-02 | 1 |
| chr9p12 | 40.2-42.4 | 8 (3.9%) | 8 (1.1%) | 1.17E-02 | 1 |
| chr9q34.3 | 136.6-140.3 | 1 (0.5%) | 40 (5.5%) | 7.29E-04 | 0.628398 |
| chr11q14.3 | 87.9-92.3 | 2 (1.0%) | 0 (0.0%) | 4.79E-02 | 1 |
| chr13q14.2 | 46.2-48.9 | 2 (1.0%) | 0 (0.0%) | 4.79E-02 | 1 |
| chr13q32.1 | 93.8-97.0 | 3 (1.5%) | 1 (0.1%) | 3.49E-02 | 1 |
| chr14q31.2 | 82.6-84.0 | 2 (1.0%) | 0 (0.0%) | 4.79E-02 | 1 |
| chr17q12 | 28.8-35.4 | 0 (0.0%) | 25 (3.4%) | 2.67E-03 | 0.954665 |
