# Supplementary material for: Copy Number Variation Analysis on a Non-Hodgkin Lymphoma Case-Control Study Identifies an 11q25 Duplication Associated with Diffuse Large B-Cell Lymphoma
Source: PLoS One. 2014 Aug 18;9(8):e105382. doi: 10.1371/journal.pone.0105382 (PMC4136881; doi:10.1371/journal.pone.0105382)
Supplement: Table S4 — Aberrations found in the CLL/SLL cases at nominal P<0.05. Aberrations that remained significant after correction (P_FDR<0.05) are shown in bold. (DOC) [file pone.0105382.s006.doc]

| **Deletions** | | | | | |
| --- | --- | --- | --- | --- | --- |
| Band | Loc (Mb, NCBI37/hg19) | Number of CLL/SLL cases (%) | Number of controls (%) | Fisher's p-value | FDR-adjusted p-value |
| chr1p35.1 | 32.2-34.4 | 2 (1.4%) | 0 (0.0%) | 2.83E-02 | 6.59E-01 |
| chr1p34.2 | 39.6-43.9 | 14 (9.5%) | 36 (4.9%) | 4.88E-02 | 9.37E-01 |
| chr2q13 | 108.6-113.8 | 4 (2.7%) | 2 (0.3%) | 8.83E-03 | 3.25E-01 |
| chr2q36.2 | 224.9-225.8 | 2 (1.4%) | 0 (0.0%) | 2.83E-02 | 6.59E-01 |
| chr3p26.1 | 5.5-8.7 | 7 (4.7%) | 9 (1.2%) | 1.00E-02 | 3.45E-01 |
| chr3q26.31 | 172.5-177.3 | 4 (2.7%) | 5 (0.7%) | 4.89E-02 | 9.37E-01 |
| chr4p16.3 | 0-3.1 | 5 (3.4%) | 2 (0.3%) | 2.01E-03 | 1.44E-01 |
| chr4p15.2 | 23.1-27.9 | 2 (1.4%) | 0 (0.0%) | 2.83E-02 | 6.59E-01 |
| chr4q13.1 | 59.2-66.3 | 34 (23.0%) | 115 (15.8%) | 4.07E-02 | 8.35E-01 |
| chr5q32 | 143.1-147.2 | 2 (1.4%) | 0 (0.0%) | 2.83E-02 | 6.59E-01 |
| chr6p22.2 | 23.5-26.1 | 2 (1.4%) | 0 (0.0%) | 2.83E-02 | 6.59E-01 |
| chr6q14.2 | 83.9-84.7 | 3 (2.0%) | 0 (0.0%) | 4.71E-03 | 2.03E-01 |
| chr6q14.3 | 84.7-87.5 | 5 (3.4%) | 3 (0.4%) | 4.64E-03 | 2.03E-01 |
| chr6q16.2 | 98.7-99.9 | 3 (2.0%) | 1 (0.1%) | 1.65E-02 | 5.27E-01 |
| chr6q16.3 | 99.9-104.8 | 6 (4.1%) | 9 (1.2%) | 2.77E-02 | 6.59E-01 |
| chr6q21 | 104.8-113.9 | 4 (2.7%) | 5 (0.7%) | 4.89E-02 | 9.37E-01 |
| chr6q22.2 | 117.1-118.6 | 3 (2.0%) | 0 (0.0%) | 4.71E-03 | 2.03E-01 |
| chr6q23.2 | 131.3-135.2 | 2 (1.4%) | 0 (0.0%) | 2.83E-02 | 6.59E-01 |
| chr6q23.3 | 135.2-139.1 | 3 (2.0%) | 2 (0.3%) | 3.62E-02 | 7.80E-01 |
| chr6q25.1 | 149.1-152.6 | 3 (2.0%) | 0 (0.0%) | 4.71E-03 | 2.03E-01 |
| chr6q25.2 | 152.6-155.6 | 2 (1.4%) | 0 (0.0%) | 2.83E-02 | 6.59E-01 |
| chr6q25.3 | 155.6-160.9 | 3 (2.0%) | 2 (0.3%) | 3.62E-02 | 7.80E-01 |
| chr8q11.23 | 52.8-55.6 | 3 (2.0%) | 0 (0.0%) | 4.71E-03 | 2.03E-01 |
| chr11q13.4 | 70.7-74.9 | 2 (1.4%) | 0 (0.0%) | 2.83E-02 | 6.59E-01 |
| chr11q14.1 | 76.7-85.3 | 6 (4.1%) | 6 (0.8%) | 7.95E-03 | 3.25E-01 |
| chr11q14.3 | 87.9-92.3 | 6 (4.1%) | 10 (1.4%) | 3.83E-02 | 8.05E-01 |
| chr11q21 | 92.3-96.7 | 4 (2.7%) | 4 (0.5%) | 3.12E-02 | 7.08E-01 |
| chr11q22.2 | 101.6-102.4 | 4 (2.7%) | 0 (0.0%) | 7.80E-04 | 6.72E-02 |
| **chr11q22.3** | **102.4-110** | **7 (4.7%)** | **1 (0.1%)** | **2.36E-05** | **4.07E-03** |
| **chr11q23.1** | **110-112.8** | **5 (3.4%)** | **0 (0.0%)** | **1.29E-04** | **1.85E-02** |
| chr11q23.2 | 112.8-115.4 | 5 (3.4%) | 1 (0.1%) | 6.66E-04 | 6.72E-02 |
| chr11q23.3 | 115.4-120.7 | 3 (2.0%) | 0 (0.0%) | 4.71E-03 | 2.03E-01 |
| chr11q24.1 | 120.7-123.5 | 3 (2.0%) | 0 (0.0%) | 4.71E-03 | 2.03E-01 |
| chr11q24.2 | 123.5-127.4 | 5 (3.4%) | 2 (0.3%) | 2.01E-03 | 1.44E-01 |
| chr13q13.3 | 34.7-39.5 | 4 (2.7%) | 0 (0.0%) | 7.80E-04 | 6.72E-02 |
| chr13q14.11 | 39.5-44.3 | 4 (2.7%) | 2 (0.3%) | 8.83E-03 | 3.25E-01 |
| chr13q14.13 | 45.9-46.2 | 4 (2.7%) | 0 (0.0%) | 7.80E-04 | 6.72E-02 |
| **chr13q14.2** | **46.2-48.9** | **8 (5.4%)** | **0 (0.0%)** | **5.55E-07** | **1.20E-04** |
| **chr13q14.3** | **48.9-52.2** | **31 (20.9%)** | **2 (0.3%)** | **2.46E-23** | **2.12E-20** |
| chr13q21.31 | 60.5-64.1 | 5 (3.4%) | 4 (0.5%) | 9.04E-03 | 3.25E-01 |
| chr13q32.3 | 98.1-100.5 | 3 (2.0%) | 0 (0.0%) | 4.71E-03 | 2.03E-01 |
| chr14q31.3 | 84-88.9 | 10 (6.8%) | 19 (2.6%) | 1.93E-02 | 5.94E-01 |
| **chr14q32.33** | **103-106.4** | **36 (24.3%)** | **34 (4.7%)** | **2.60E-12** | **1.12E-09** |
| chr20p11.21 | 22.3-25.7 | 3 (2.0%) | 1 (0.1%) | 1.65E-02 | 5.27E-01 |
| **chr22q11.22** | **20.5-21.8** | **10 (6.8%)** | **1 (0.1%)** | **1.34E-07** | **3.85E-05** |
| **Duplications** | | | | | |
| Band | Loc (Mb, NCBI37/hg19) | Number of CLL/SLL cases (%) | Number of controls (%) | Fisher's p-value | FDR-adjusted p-value |
| chr2p25.3 | 0-4.3 | 3 (2.0%) | 1 (0.1%) | 1.65E-02 | 0.938253846 |
| chr2p21 | 41.6-47.6 | 4 (2.7%) | 4 (0.5%) | 3.12E-02 | 0.996088889 |
| chr2p16.1 | 54.8-61.1 | 6 (4.1%) | 7 (1.0%) | 1.27E-02 | 0.938253846 |
| chr5q12.1 | 58.9-63.0 | 3 (2.0%) | 1 (0.1%) | 1.65E-02 | 0.938253846 |
| chr6p25.1 | 4.1-7 | 2 (1.4%) | 0 (0.0%) | 2.83E-02 | 0.938253846 |
| chr6p24.1 | 11.2-13.5 | 2 (1.4%) | 0 (0.0%) | 2.83E-02 | 0.938253846 |
| chr6q16.3 | 99.9-104.8 | 2 (1.4%) | 0 (0.0%) | 2.83E-02 | 0.938253846 |
| chr6q22.31 | 118.6-126.2 | 3 (2.0%) | 1 (0.1%) | 1.65E-02 | 0.938253846 |
| chr8q23.1 | 106.1-110.6 | 2 (1.4%) | 0 (0.0%) | 2.83E-02 | 0.938253846 |
| chr11q14.3 | 87.9-92.3 | 2 (1.4%) | 0 (0.0%) | 2.83E-02 | 0.938253846 |
| chr12p13.33 | 0-3.1 | 3 (2.0%) | 2 (0.3%) | 3.62E-02 | 1 |
| chr12p12.3 | 14.8-19.9 | 6 (4.1%) | 9 (1.2%) | 2.77E-02 | 0.938253846 |
| chr12p12.2 | 19.9-21.2 | 2 (1.4%) | 0 (0.0%) | 2.83E-02 | 0.938253846 |
| chr12p11.23 | 26.3-27.7 | 2 (1.4%) | 0 (0.0%) | 2.83E-02 | 0.938253846 |
| chr12p11.22 | 27.7-30.6 | 3 (2.0%) | 0 (0.0%) | 4.71E-03 | 0.938253846 |
| chr12q14.1 | 56.3-61.4 | 3 (2.0%) | 1 (0.1%) | 1.65E-02 | 0.938253846 |
| chr12q15 | 66-69.8 | 2 (1.4%) | 0 (0.0%) | 2.83E-02 | 0.938253846 |
| chr12q21.1 | 69.8-74.1 | 2 (1.4%) | 0 (0.0%) | 2.83E-02 | 0.938253846 |
| chr12q21.31 | 78.7-85.1 | 5 (3.4%) | 5 (0.7%) | 1.56E-02 | 0.938253846 |
| chr12q21.32 | 85.1-87.5 | 2 (1.4%) | 0 (0.0%) | 2.83E-02 | 0.938253846 |
| chr12q23.1 | 94.8-100 | 3 (2.0%) | 0 (0.0%) | 4.71E-03 | 0.938253846 |
| chr12q23.3 | 102.4-107.5 | 2 (1.4%) | 0 (0.0%) | 2.83E-02 | 0.938253846 |
| chr12q24.13 | 110.8-112.8 | 2 (1.4%) | 0 (0.0%) | 2.83E-02 | 0.938253846 |
| chr12q24.23 | 116.7-119.1 | 2 (1.4%) | 0 (0.0%) | 2.83E-02 | 0.938253846 |
| chr12q24.31 | 119.1-124.5 | 3 (2.0%) | 0 (0.0%) | 4.71E-03 | 0.938253846 |
| chr13q21.1 | 52.2-57.6 | 4 (2.7%) | 2 (0.3%) | 8.83E-03 | 0.938253846 |
| chr17p13.1 | 6.8-11.2 | 3 (2.0%) | 1 (0.1%) | 1.65E-02 | 0.938253846 |
| chr19p12 | 19.8-26.7 | 5 (3.4%) | 6 (0.8%) | 2.48E-02 | 0.938253846 |
