# Supplementary material for: Copy Number Variation Analysis on a Non-Hodgkin Lymphoma Case-Control Study Identifies an 11q25 Duplication Associated with Diffuse Large B-Cell Lymphoma
Source: PLoS One. 2014 Aug 18;9(8):e105382. doi: 10.1371/journal.pone.0105382 (PMC4136881; doi:10.1371/journal.pone.0105382)
Supplement: Table S5 — Genes significantly deleted in the CLL/SLL cases (FDR p-value<0.05). (DOC) [file pone.0105382.s007.doc]

| Gene | Loc (NCB136/hg18) | Number of CLL/SLL cases (%) | Number of controls (%) | Fisher's p-value | FDR-adjusted p-value |
| --- | --- | --- | --- | --- | --- |
| DLEU2 | 13:49454688-49597678 | 27 (18.2%) | 1 (0.1%) | 3.83E-21 | 2.24E-17 |
| ST13P4 | 13:49644154-49645752 | 23 (15.5%) | 0 (0.0%) | 3.62E-19 | 7.04E-16 |
| DLEU1 | 13:49554414-49577434 | 23 (15.5%) | 0 (0.0%) | 3.62E-19 | 7.04E-16 |
| MIR16-1 | 13:49521109-49521198 | 24 (16.2%) | 1 (0.1%) | 1.14E-18 | 1.33E-15 |
| MIR15A | 13:49521255-49521338 | 24 (16.2%) | 1 (0.1%) | 1.14E-18 | 1.33E-15 |
| DLEU7 | 13:50184759-50315886 | 22 (14.9%) | 0 (0.0%) | 2.46E-18 | 2.05E-15 |
| DLEU7-AS1 | 13:50279991-50322042 | 22 (14.9%) | 0 (0.0%) | 2.46E-18 | 2.05E-15 |
| RNASEH2B-AS1 | 13:50354515-50382849 | 21 (14.2%) | 0 (0.0%) | 1.66E-17 | 1.21E-14 |
| RNASEH2B | 13:50381814-50442597 | 18 (12.2%) | 0 (0.0%) | 4.90E-15 | 3.18E-12 |
| TRIM13 | 13:49469143-49490604 | 14 (9.5%) | 0 (0.0%) | 8.80E-12 | 4.28E-09 |
| KCNRG | 13:49487390-49493059 | 14 (9.5%) | 0 (0.0%) | 8.80E-12 | 4.28E-09 |
| MIR3613 | 13:49468551-49468638 | 14 (9.5%) | 0 (0.0%) | 8.80E-12 | 4.28E-09 |
| LINC00221 | 14:106009499-106022574 | 13 (8.8%) | 0 (0.0%) | 5.64E-11 | 2.53E-08 |
| GUCY1B2 | 13:50466647-50538294 | 12 (8.1%) | 0 (0.0%) | 3.59E-10 | 1.50E-07 |
| LINC00226 | 14:105815313-105816011 | 21 (14.2%) | 13 (1.8%) | 2.10E-09 | 8.17E-07 |
| RCBTB1 | 13:49004082-49057720 | 10 (6.8%) | 0 (0.0%) | 1.43E-08 | 4.39E-06 |
| SETDB2 | 13:48916429-48967140 | 10 (6.8%) | 0 (0.0%) | 1.43E-08 | 4.39E-06 |
| PHF11 | 13:48967801-49001118 | 10 (6.8%) | 0 (0.0%) | 1.43E-08 | 4.39E-06 |
| KPNA3 | 13:49171443-49265058 | 10 (6.8%) | 0 (0.0%) | 1.43E-08 | 4.39E-06 |
| EBPL | 13:49132810-49163624 | 9 (6.1%) | 0 (0.0%) | 8.93E-08 | 2.08E-05 |
| FAM124A | 13:50694470-50756378 | 9 (6.1%) | 0 (0.0%) | 8.93E-08 | 2.08E-05 |
| SPRYD7 | 13:49384842-49408626 | 9 (6.1%) | 0 (0.0%) | 8.93E-08 | 2.08E-05 |
| SERPINE3 | 13:50813168-50834240 | 9 (6.1%) | 0 (0.0%) | 8.93E-08 | 2.08E-05 |
| ADAM6 | 14:105506863-105509403 | 9 (6.1%) | 0 (0.0%) | 8.93E-08 | 2.08E-05 |
| KIAA0125 | 14:105454882-105469547 | 9 (6.1%) | 0 (0.0%) | 8.93E-08 | 2.08E-05 |
| FNDC3A | 13:48448048-48681916 | 8 (5.4%) | 0 (0.0%) | 5.55E-07 | 9.53E-05 |
| CAB39L | 13:48780786-48916222 | 8 (5.4%) | 0 (0.0%) | 5.55E-07 | 9.53E-05 |
| INTS6 | 13:50833701-50925276 | 8 (5.4%) | 0 (0.0%) | 5.55E-07 | 9.53E-05 |
| DHRS12 | 13:51240129-51276299 | 8 (5.4%) | 0 (0.0%) | 5.55E-07 | 9.53E-05 |
| WDFY2 | 13:51056484-51234172 | 8 (5.4%) | 0 (0.0%) | 5.55E-07 | 9.53E-05 |
| ARL11 | 13:49100435-49106009 | 8 (5.4%) | 0 (0.0%) | 5.55E-07 | 9.53E-05 |
| CTAGE10P | 13:49362545-49365517 | 8 (5.4%) | 0 (0.0%) | 5.55E-07 | 9.53E-05 |
| MIR5693 | 13:50820703-50820776 | 8 (5.4%) | 0 (0.0%) | 5.55E-07 | 9.53E-05 |
| MIR4703 | 13:51024725-51024804 | 8 (5.4%) | 0 (0.0%) | 5.55E-07 | 9.53E-05 |
| CDADC1 | 13:48720047-48765623 | 7 (4.7%) | 0 (0.0%) | 3.43E-06 | 5.27E-04 |
| MLNR | 13:48692474-48694514 | 7 (4.7%) | 0 (0.0%) | 3.43E-06 | 5.27E-04 |
| LINC00282 | 13:51285483-51317287 | 7 (4.7%) | 0 (0.0%) | 3.43E-06 | 5.27E-04 |
| MIR650 | 22:21495269-21495365 | 7 (4.7%) | 0 (0.0%) | 3.43E-06 | 5.27E-04 |
| CCDC70 | 13:51334117-51338373 | 6 (4.1%) | 0 (0.0%) | 2.11E-05 | 3.16E-03 |
| DYNC2H1 | 11:102485369-102855801 | 5 (3.4%) | 0 (0.0%) | 1.29E-04 | 6.27E-03 |
| GRIA4 | 11:104986009-105358029 | 5 (3.4%) | 0 (0.0%) | 1.29E-04 | 6.27E-03 |
| GUCY1A2 | 11:106049947-106394381 | 5 (3.4%) | 0 (0.0%) | 1.29E-04 | 6.27E-03 |
| CWF19L2 | 11:106702281-106833782 | 5 (3.4%) | 0 (0.0%) | 1.29E-04 | 6.27E-03 |
| SLC35F2 | 11:107166926-107235124 | 5 (3.4%) | 0 (0.0%) | 1.29E-04 | 6.27E-03 |
| SLN | 11:107083310-107087997 | 5 (3.4%) | 0 (0.0%) | 1.29E-04 | 6.27E-03 |
| CUL5 | 11:107384617-107483698 | 5 (3.4%) | 0 (0.0%) | 1.29E-04 | 6.27E-03 |
| KDELC2 | 11:107848042-107874369 | 5 (3.4%) | 0 (0.0%) | 1.29E-04 | 6.27E-03 |
| ATM | 11:107598768-107745036 | 5 (3.4%) | 0 (0.0%) | 1.29E-04 | 6.27E-03 |
| DDX10 | 11:108041025-108316858 | 5 (3.4%) | 0 (0.0%) | 1.29E-04 | 6.27E-03 |
| FDX1 | 11:109805870-109840818 | 5 (3.4%) | 0 (0.0%) | 1.29E-04 | 6.27E-03 |
| ARHGAP20 | 11:109952968-110089122 | 5 (3.4%) | 0 (0.0%) | 1.29E-04 | 6.27E-03 |
| RDX | 11:109550814-109672647 | 5 (3.4%) | 0 (0.0%) | 1.29E-04 | 6.27E-03 |
| BTG4 | 11:110843465-110888289 | 5 (3.4%) | 0 (0.0%) | 1.29E-04 | 6.27E-03 |
| SIK2 | 11:110978379-111102842 | 5 (3.4%) | 0 (0.0%) | 1.29E-04 | 6.27E-03 |
| DLAT | 11:111400747-111440212 | 5 (3.4%) | 0 (0.0%) | 1.29E-04 | 6.27E-03 |
| ZW10 | 11:113109114-113149695 | 5 (3.4%) | 0 (0.0%) | 1.29E-04 | 6.27E-03 |
| NCAM1 | 11:112337178-112654368 | 5 (3.4%) | 0 (0.0%) | 1.29E-04 | 6.27E-03 |
| TTC12 | 11:112690460-112742324 | 5 (3.4%) | 0 (0.0%) | 1.29E-04 | 6.27E-03 |
| NXPE1 | 11:113897646-113935790 | 5 (3.4%) | 0 (0.0%) | 1.29E-04 | 6.27E-03 |
| ZBTB16 | 11:113435640-113626607 | 5 (3.4%) | 0 (0.0%) | 1.29E-04 | 6.27E-03 |
| CADM1 | 11:114549554-114880451 | 5 (3.4%) | 0 (0.0%) | 1.29E-04 | 6.27E-03 |
| MSANTD4 | 11:105383838-105398164 | 5 (3.4%) | 0 (0.0%) | 1.29E-04 | 6.27E-03 |
| KBTBD3 | 11:105427034-105453675 | 5 (3.4%) | 0 (0.0%) | 1.29E-04 | 6.27E-03 |
| AASDHPPT | 11:105453501-105474629 | 5 (3.4%) | 0 (0.0%) | 1.29E-04 | 6.27E-03 |
| ALKBH8 | 11:106878662-106941671 | 5 (3.4%) | 0 (0.0%) | 1.29E-04 | 6.27E-03 |
| ELMOD1 | 11:106967026-107042715 | 5 (3.4%) | 0 (0.0%) | 1.29E-04 | 6.27E-03 |
| LOC643923 | 11:106967680-106969159 | 5 (3.4%) | 0 (0.0%) | 1.29E-04 | 6.27E-03 |
| RAB39A | 11:107304486-107339418 | 5 (3.4%) | 0 (0.0%) | 1.29E-04 | 6.27E-03 |
| NPAT | 11:107533327-107598575 | 5 (3.4%) | 0 (0.0%) | 1.29E-04 | 6.27E-03 |
| ACAT1 | 11:107497467-107524101 | 5 (3.4%) | 0 (0.0%) | 1.29E-04 | 6.27E-03 |
| C11orf65 | 11:107758936-107843468 | 5 (3.4%) | 0 (0.0%) | 1.29E-04 | 6.27E-03 |
| EXPH5 | 11:107881367-107969584 | 5 (3.4%) | 0 (0.0%) | 1.29E-04 | 6.27E-03 |
| C11orf87 | 11:108798055-108805103 | 5 (3.4%) | 0 (0.0%) | 1.29E-04 | 6.27E-03 |
| ZC3H12C | 11:109469296-109547776 | 5 (3.4%) | 0 (0.0%) | 1.29E-04 | 6.27E-03 |
| C11orf93 | 11:110674480-110684670 | 5 (3.4%) | 0 (0.0%) | 1.29E-04 | 6.27E-03 |
| POU2AF1 | 11:110728189-110755367 | 5 (3.4%) | 0 (0.0%) | 1.29E-04 | 6.27E-03 |
| C11orf53 | 11:110631916-110662182 | 5 (3.4%) | 0 (0.0%) | 1.29E-04 | 6.27E-03 |
| MIR4491 | 11:110723691-110723757 | 5 (3.4%) | 0 (0.0%) | 1.29E-04 | 6.27E-03 |
| C11orf92 | 11:110669323-110675749 | 5 (3.4%) | 0 (0.0%) | 1.29E-04 | 6.27E-03 |
| LOC100132078 | 11:110790176-110794121 | 5 (3.4%) | 0 (0.0%) | 1.29E-04 | 6.27E-03 |
| C11orf88 | 11:110890719-110912966 | 5 (3.4%) | 0 (0.0%) | 1.29E-04 | 6.27E-03 |
| LAYN | 11:110916442-110937680 | 5 (3.4%) | 0 (0.0%) | 1.29E-04 | 6.27E-03 |
| MIR34C | 11:110889373-110889450 | 5 (3.4%) | 0 (0.0%) | 1.29E-04 | 6.27E-03 |
| MIR34B | 11:110888872-110888956 | 5 (3.4%) | 0 (0.0%) | 1.29E-04 | 6.27E-03 |
| PPP2R1B | 11:111102841-111142379 | 5 (3.4%) | 0 (0.0%) | 1.29E-04 | 6.27E-03 |
| ALG9 | 11:111158128-111247515 | 5 (3.4%) | 0 (0.0%) | 1.29E-04 | 6.27E-03 |
| C11orf1 | 11:111255157-111260007 | 5 (3.4%) | 0 (0.0%) | 1.29E-04 | 6.27E-03 |
| FDXACB1 | 11:111249989-111255391 | 5 (3.4%) | 0 (0.0%) | 1.29E-04 | 6.27E-03 |
| HSPB2-C11orf52 | 11:111288669-111302805 | 5 (3.4%) | 0 (0.0%) | 1.29E-04 | 6.27E-03 |
| CRYAB | 11:111284559-111287683 | 5 (3.4%) | 0 (0.0%) | 1.29E-04 | 6.27E-03 |
| HSPB2 | 11:111288669-111290027 | 5 (3.4%) | 0 (0.0%) | 1.29E-04 | 6.27E-03 |
| DIXDC1 | 11:111313136-111398517 | 5 (3.4%) | 0 (0.0%) | 1.29E-04 | 6.27E-03 |
| C11orf52 | 11:111294810-111302805 | 5 (3.4%) | 0 (0.0%) | 1.29E-04 | 6.27E-03 |
| PIH1D2 | 11:111439943-111450105 | 5 (3.4%) | 0 (0.0%) | 1.29E-04 | 6.27E-03 |
| SDHD | 11:111462757-111471735 | 5 (3.4%) | 0 (0.0%) | 1.29E-04 | 6.27E-03 |
| C11orf57 | 11:111450177-111461084 | 5 (3.4%) | 0 (0.0%) | 1.29E-04 | 6.27E-03 |
| IL18 | 11:111519183-111540050 | 5 (3.4%) | 0 (0.0%) | 1.29E-04 | 6.27E-03 |
| TIMM8B | 11:111460745-111462732 | 5 (3.4%) | 0 (0.0%) | 1.29E-04 | 6.27E-03 |
| TEX12 | 11:111543304-111548489 | 5 (3.4%) | 0 (0.0%) | 1.29E-04 | 6.27E-03 |
| BCO2 | 11:111551417-111594859 | 5 (3.4%) | 0 (0.0%) | 1.29E-04 | 6.27E-03 |
| PTS | 11:111602297-111609905 | 5 (3.4%) | 0 (0.0%) | 1.29E-04 | 6.27E-03 |
| LOC100288346 | 11:112645463-112649833 | 5 (3.4%) | 0 (0.0%) | 1.29E-04 | 6.27E-03 |
| DRD2 | 11:112785526-112851211 | 5 (3.4%) | 0 (0.0%) | 1.29E-04 | 6.27E-03 |
| MIR4301 | 11:112825954-112826020 | 5 (3.4%) | 0 (0.0%) | 1.29E-04 | 6.27E-03 |
| ANKK1 | 11:112763722-112776350 | 5 (3.4%) | 0 (0.0%) | 1.29E-04 | 6.27E-03 |
| TMPRSS5 | 11:113063477-113082278 | 5 (3.4%) | 0 (0.0%) | 1.29E-04 | 6.27E-03 |
| CLDN25 | 11:113155727-113156417 | 5 (3.4%) | 0 (0.0%) | 1.29E-04 | 6.27E-03 |
| HTR3A | 11:113351006-113366244 | 5 (3.4%) | 0 (0.0%) | 1.29E-04 | 6.27E-03 |
| HTR3B | 11:113280727-113322493 | 5 (3.4%) | 0 (0.0%) | 1.29E-04 | 6.27E-03 |
| NNMT | 11:113671744-113688448 | 5 (3.4%) | 0 (0.0%) | 1.29E-04 | 6.27E-03 |
| REXO2 | 11:113815317-113826210 | 5 (3.4%) | 0 (0.0%) | 1.29E-04 | 6.27E-03 |
| NXPE4 | 11:113946522-113971694 | 5 (3.4%) | 0 (0.0%) | 1.29E-04 | 6.27E-03 |
| NXPE2 | 11:114054409-114082862 | 5 (3.4%) | 0 (0.0%) | 1.29E-04 | 6.27E-03 |
| LINC00462 | 13:48049110-48053038 | 5 (3.4%) | 0 (0.0%) | 1.29E-04 | 6.27E-03 |
| CYSLTR2 | 13:48178951-48181499 | 5 (3.4%) | 0 (0.0%) | 1.29E-04 | 6.27E-03 |
| ATP7B | 13:51404805-51483631 | 5 (3.4%) | 0 (0.0%) | 1.29E-04 | 6.27E-03 |
| ALG11 | 13:51484523-51501781 | 5 (3.4%) | 0 (0.0%) | 1.29E-04 | 6.27E-03 |
| UTP14C | 13:51496827-51505737 | 5 (3.4%) | 0 (0.0%) | 1.29E-04 | 6.27E-03 |
| NEK3 | 13:51604779-51631997 | 5 (3.4%) | 0 (0.0%) | 1.29E-04 | 6.27E-03 |
| NEK5 | 13:51536900-51601215 | 5 (3.4%) | 0 (0.0%) | 1.29E-04 | 6.27E-03 |
| BIRC3 | 11:101693390-101715344 | 4 (2.7%) | 0 (0.0%) | 7.80E-04 | 2.46E-02 |
| USP28 | 11:113173806-113251466 | 4 (2.7%) | 0 (0.0%) | 7.80E-04 | 2.46E-02 |
| SIK3 | 11:116219327-116474203 | 4 (2.7%) | 0 (0.0%) | 7.80E-04 | 2.46E-02 |
| ARHGAP42 | 11:100063616-100366866 | 4 (2.7%) | 0 (0.0%) | 7.80E-04 | 2.46E-02 |
| LOC101054525 | 11:100505017-100535211 | 4 (2.7%) | 0 (0.0%) | 7.80E-04 | 2.46E-02 |
| C11orf70 | 11:101423378-101460501 | 4 (2.7%) | 0 (0.0%) | 7.80E-04 | 2.46E-02 |
| TRPC6 | 11:100827504-100959869 | 4 (2.7%) | 0 (0.0%) | 7.80E-04 | 2.46E-02 |
| KIAA1377 | 11:101290955-101377003 | 4 (2.7%) | 0 (0.0%) | 7.80E-04 | 2.46E-02 |
| YAP1 | 11:101486401-101609364 | 4 (2.7%) | 0 (0.0%) | 7.80E-04 | 2.46E-02 |
| MMP20 | 11:101952775-102001273 | 4 (2.7%) | 0 (0.0%) | 7.80E-04 | 2.46E-02 |
| PDGFD | 11:103283123-103540237 | 4 (2.7%) | 0 (0.0%) | 7.80E-04 | 2.46E-02 |
| CASP4 | 11:104318803-104344535 | 4 (2.7%) | 0 (0.0%) | 7.80E-04 | 2.46E-02 |
| C11orf71 | 11:113767379-113776482 | 4 (2.7%) | 0 (0.0%) | 7.80E-04 | 2.46E-02 |
| BUD13 | 11:116124095-116148924 | 4 (2.7%) | 0 (0.0%) | 7.80E-04 | 2.46E-02 |
| DSCAML1 | 11:116803698-117173186 | 4 (2.7%) | 0 (0.0%) | 7.80E-04 | 2.46E-02 |
| PAFAH1B2 | 11:116520209-116552802 | 4 (2.7%) | 0 (0.0%) | 7.80E-04 | 2.46E-02 |
| CEP164 | 11:116697703-116789192 | 4 (2.7%) | 0 (0.0%) | 7.80E-04 | 2.46E-02 |
| RNF214 | 11:116608613-116661614 | 4 (2.7%) | 0 (0.0%) | 7.80E-04 | 2.46E-02 |
| MIR548G | 11:100288165-100288199 | 4 (2.7%) | 0 (0.0%) | 7.80E-04 | 2.46E-02 |
| TMEM133 | 11:100368020-100369876 | 4 (2.7%) | 0 (0.0%) | 7.80E-04 | 2.46E-02 |
| PGR | 11:100405564-100505754 | 4 (2.7%) | 0 (0.0%) | 7.80E-04 | 2.46E-02 |
| MIR3920 | 11:100895760-100895846 | 4 (2.7%) | 0 (0.0%) | 7.80E-04 | 2.46E-02 |
| ANGPTL5 | 11:101266614-101292463 | 4 (2.7%) | 0 (0.0%) | 7.80E-04 | 2.46E-02 |
| BIRC2 | 11:101723122-101754611 | 4 (2.7%) | 0 (0.0%) | 7.80E-04 | 2.46E-02 |
| TMEM123 | 11:101772265-101828985 | 4 (2.7%) | 0 (0.0%) | 7.80E-04 | 2.46E-02 |
| MMP7 | 11:101896448-101906688 | 4 (2.7%) | 0 (0.0%) | 7.80E-04 | 2.46E-02 |
| MMP27 | 11:102067624-102081678 | 4 (2.7%) | 0 (0.0%) | 7.80E-04 | 2.46E-02 |
| MMP8 | 11:102087735-102100895 | 4 (2.7%) | 0 (0.0%) | 7.80E-04 | 2.46E-02 |
| MMP1 | 11:102165850-102174176 | 4 (2.7%) | 0 (0.0%) | 7.80E-04 | 2.46E-02 |
| MMP3 | 11:102211737-102219552 | 4 (2.7%) | 0 (0.0%) | 7.80E-04 | 2.46E-02 |
| MMP10 | 11:102146442-102156569 | 4 (2.7%) | 0 (0.0%) | 7.80E-04 | 2.46E-02 |
| WTAPP1 | 11:102159616-102212707 | 4 (2.7%) | 0 (0.0%) | 7.80E-04 | 2.46E-02 |
| MMP13 | 11:102318930-102331673 | 4 (2.7%) | 0 (0.0%) | 7.80E-04 | 2.46E-02 |
| MMP12 | 11:102238673-102250974 | 4 (2.7%) | 0 (0.0%) | 7.80E-04 | 2.46E-02 |
| DCUN1D5 | 11:102426622-102468154 | 4 (2.7%) | 0 (0.0%) | 7.80E-04 | 2.46E-02 |
| MIR4693 | 11:103225843-103225918 | 4 (2.7%) | 0 (0.0%) | 7.80E-04 | 2.46E-02 |
| DDI1 | 11:103412517-103415132 | 4 (2.7%) | 0 (0.0%) | 7.80E-04 | 2.46E-02 |
| LOC643733 | 11:104277485-104294112 | 4 (2.7%) | 0 (0.0%) | 7.80E-04 | 2.46E-02 |
| CASP12 | 11:104261654-104274607 | 4 (2.7%) | 0 (0.0%) | 7.80E-04 | 2.46E-02 |
| CARD16 | 11:104417262-104421261 | 4 (2.7%) | 0 (0.0%) | 7.80E-04 | 2.46E-02 |
| CASP1 | 11:104401446-104411094 | 4 (2.7%) | 0 (0.0%) | 7.80E-04 | 2.46E-02 |
| CASP5 | 11:104370176-104399105 | 4 (2.7%) | 0 (0.0%) | 7.80E-04 | 2.46E-02 |
| CARD18 | 11:104513657-104515671 | 4 (2.7%) | 0 (0.0%) | 7.80E-04 | 2.46E-02 |
| CARD17 | 11:104468405-104477368 | 4 (2.7%) | 0 (0.0%) | 7.80E-04 | 2.46E-02 |
| C11orf34 | 11:111624085-111636793 | 4 (2.7%) | 0 (0.0%) | 7.80E-04 | 2.46E-02 |
| RBM7 | 11:113776593-113784845 | 4 (2.7%) | 0 (0.0%) | 7.80E-04 | 2.46E-02 |
| LOC283143 | 11:115131258-115136128 | 4 (2.7%) | 0 (0.0%) | 7.80E-04 | 2.46E-02 |
| APOA5 | 11:116165295-116168346 | 4 (2.7%) | 0 (0.0%) | 7.80E-04 | 2.46E-02 |
| APOA1 | 11:116211678-116213548 | 4 (2.7%) | 0 (0.0%) | 7.80E-04 | 2.46E-02 |
| APOC3 | 11:116205833-116208997 | 4 (2.7%) | 0 (0.0%) | 7.80E-04 | 2.46E-02 |
| APOA4 | 11:116196627-116199221 | 4 (2.7%) | 0 (0.0%) | 7.80E-04 | 2.46E-02 |
| ZNF259 | 11:116154485-116163949 | 4 (2.7%) | 0 (0.0%) | 7.80E-04 | 2.46E-02 |
| PCSK7 | 11:116580997-116608021 | 4 (2.7%) | 0 (0.0%) | 7.80E-04 | 2.46E-02 |
| TAGLN | 11:116575249-116580718 | 4 (2.7%) | 0 (0.0%) | 7.80E-04 | 2.46E-02 |
| LOC100652768 | 11:116571538-116577840 | 4 (2.7%) | 0 (0.0%) | 7.80E-04 | 2.46E-02 |
| SIDT2 | 11:116555148-116573371 | 4 (2.7%) | 0 (0.0%) | 7.80E-04 | 2.46E-02 |
| BACE1 | 11:116661611-116692182 | 4 (2.7%) | 0 (0.0%) | 7.80E-04 | 2.46E-02 |
| BACE1-AS | 11:116667271-116668067 | 4 (2.7%) | 0 (0.0%) | 7.80E-04 | 2.46E-02 |
| ITM2B | 13:47705274-47734233 | 4 (2.7%) | 0 (0.0%) | 7.80E-04 | 2.46E-02 |
| RCBTB2 | 13:47961099-48005317 | 4 (2.7%) | 0 (0.0%) | 7.80E-04 | 2.46E-02 |
| MRPS31P5 | 13:51639845-51666603 | 4 (2.7%) | 0 (0.0%) | 7.80E-04 | 2.46E-02 |
| LOC648691 | 22:21231755-21239007 | 4 (2.7%) | 0 (0.0%) | 7.80E-04 | 2.46E-02 |
| ZNF280B | 22:21168771-21193505 | 4 (2.7%) | 0 (0.0%) | 7.80E-04 | 2.46E-02 |
| ZNF280A | 22:21198060-21204613 | 4 (2.7%) | 0 (0.0%) | 7.80E-04 | 2.46E-02 |
| PRAME | 22:21220122-21231696 | 4 (2.7%) | 0 (0.0%) | 7.80E-04 | 2.46E-02 |
